# Safety and Efficacy of SARS-CoV-2 Vaccines in Patients With Chronic Liver Diseases: A Systematic Review and Meta-Analysis

**DOI:** 10.3389/ijph.2024.1605295

**Published:** 2024-11-21

**Authors:** Guanglin Xiao, Taiyu He, Biqiong Zhang, Ziqiao Yang, Ning Ling, Min Chen, Dazhi Zhang, Peng Hu, Gaoli Zhang, Mingli Peng, Dachuan Cai, Hong Ren

**Affiliations:** Department of Infectious Diseases, Key Laboratory of Molecular Biology for Infectious Diseases, Ministry of Education, Institute for Viral Hepatitis, The Second Affiliated Hospital, Chongqing Medical University, Chongqing, China

**Keywords:** vaccine, meta-analysis, safety, efficacy, chronic liver disease

## Abstract

**Objectives:**

This review aimed to assess the safety and efficacy of SARS-CoV-2 vaccines in patients with chronic liver disease (CLD).

**Methods:**

Cochrane Central Register of Controlled Trials, PubMed, Embase, and Web of Science were searched from 2020 to 2024. Data was extracted following Preferred Reporting Items for Systematic Review and Meta-Analyses guidelines. The random-effects model (when I^2^ ≥ 50%) or fixed effect model (I^2^ < 50%) was used.

**Results:**

29 studies were included in this review. Compared to healthy controls (HCs), patients with CLD had a higher incidence of mild adverse events (RR = 1.60, *p* < 0.001), while the incidence of severe adverse events was similar (RR = 1.08, *p* = 0.92). Seropositivity rates of three antibodies in patients were lower than in HCs [neutralizing antibody (RR = 0.86, *p* = 0.002), anti-spike antibody (RR = 0.97, *p* = 0.06) and anti-receptor binding domain antibody (RR = 0.95, *p* = 0.04)]. Compared to unvaccinated patients, vaccinated patients had lower rates of SARS-CoV-2 infection, hospitalization and death (*p* ≤ 0.05).

**Conclusion:**

SARS-CoV-2 vaccines showed good safety and efficacy in CLD patients, but antibody response appeared to be decreased. Therefore, SARS-CoV-2 vaccines and booster doses should be given priority in this vulnerable population.

## Introduction

The rapid development and deployment of vaccinations against SARS-CoV-2, alongside a degree of naturally acquired immunity from past infection, has transformed the landscape of the COVID-19 pandemic. At a population level, vaccination has been shown to reduce SARS-CoV-2 infection and protect against hospitalisation and death from severe COVID-19. However, understanding the immunogenicity and effectiveness of vaccination programmes in vulnerable cohorts with chronic disease remains an important clinical priority [1]. Patients with liver diseases might have worse outcome from COVID-19 than the general population [2–4]. Fortunately, vaccination is effective in preventing SARS-CoV-2 infection, severe symptom and death [5–7]. And societies in Europe, United States and China have recommended SARS-CoV-2 vaccination of all patients with CLD [8–10]. However, previous large cohort clinical trials of SARS-CoV-2 vaccines only included a few patients with CLD [11–13], and did not show the separate results of these patients. To our knowledge, studies on the safety and efficacy of SARS-CoV-2 vaccines in patients with CLD were still limited, and varied in populations, vaccine types and results. So, there is a need to further explore the safety and efficacy of SARS-CoV-2 vaccines in patients with CLD.

This systematic review and meta-analysis was performed to better understand the safety and efficacy of SARS-CoV-2 vaccines in patients with CLD, and it may be helpful for clinical practice.

## Methods

### Protocol and Registration

This systematic review and meta-analysis was conducted following a pre-established protocol according to the Preferred Reporting Items for Systematic Review and Meta-Analyses guidelines [14]. The protocol was initially registered in PROSPERO (registration number CRD42022302993) on 12 January 2022 [15].

### Eligibility Criteria

Studies were eligible for being included in this systematic review and meta-analysis if they met the following criteria: 1) study included at least 20 adults aged ≥18 years with chronic liver disease of any severity or etiology (liver transplantation recipients were excluded) with/without COVID-19; 2) intervention was full-course vaccination (one dose: Johnson & Johnson, Cansino; two doses: other type of SARS-CoV-2 vaccines) of any type of SARS-CoV-2 vaccine with specific interval time; 3) intervention was compared with placebo, other vaccines or no vaccination; 4) outcomes included incidence of mild adverse events (MAEs), or incidence of severe adverse events (SAEs), or seropositivity/seroconversion rates of antibodies against SARS-CoV-2, or SARS-CoV-2 infection, or COVID-19-related hospitalization, or COVID-19-related mortality; 5) study type was randomized or non-randomized controlled trial, or cohort study, or case-control study, or cross-sectional study.

We included studies published in any kind of language. Review articles, case reports, animal studies, editorials, clinical guidelines, comment, meeting abstract, studies on CLD patients but only including liver transplant recipients (response to the vaccination and clinical outcomes are likely to be strongly influenced by the immunosuppressive medication rather than the status of liver disease), studies without separate outcomes of patients with chronic liver diseases, and studies retracted from publication were excluded.

### Study Identification

We searched the Cochrane Central Register of Controlled Trials, PubMed, Embase, and Web of Science (from 2020 to 1 June 2024) for relevant articles. The Medical Subject Headings (MeSH) terms and free-text terms used were as follows: liver diseases, hepatic diseases, chronic liver diseases, cirrhosis, hepatitis, NAFLD, alcoholic liver disease, COVID-19, COVID-19 vaccines, SARS-CoV-2, vaccine, vaccination, immunization. Combination of these MeSH terms and free-text terms were used in each database. Relevant reviews and the reference list of the included articles were also checked to search for additional studies. The detailed searching strategies are shown in Supplementary Table S1.

### Study Selection

Titles and abstracts of all articles were screened by two independent reviewers to assess whether they met inclusion criteria. Studies deemed eligible were then included in the full-text review by two independent reviewers. Disagreements were resolved by discussion or consulting a third reviewer, and the reasons for exclusion were recorded.

### Data Extraction and Quality Assessment

The data were extracted by two independent reviewers and saved in a standardized form. Data extracted include the follows: participants (the number of participants, demographic and clinical characteristics), interventions and comparators (vaccine type, dose, comparator type, number of participants in intervention and comparison group, follow-up time after full-course vaccination), outcomes (the outcomes mentioned above, the unit of outcome), study designs (study type, location, date), study quality and study bias, other information: authors, publication time, etc.

The Newcastle-Ottawa Scale [16] was used to assess the quality of cohort study, and based on the total scores, cohort studies were classified as having low (7–9 stars), moderate (5–6 stars), and high (1–4 stars) risk of bias, respectively. The checklist recommended by Agency for Healthcare Research and Quality (AHRQ) [17] was used to assess the quality of cross-sectional study, and for each item of the checklist, 1 point (answered “yes”) or 0 point (answered “no” or “unclear”) was assigned. Based on the total scores, cross-sectional studies were classified as having low [8–11], moderate [4–7], and high (0–3) risk of bias, respectively. The assessment was completed by two reviewers independently, and the discrepancy was resolved through discussion or consulting a third reviewer.

### Data Synthesis and Statistical Analysis

The safety outcomes were the incidence of MAEs, and incidence of SAEs. The efficacy outcomes were seropositivity/seroconversion rates of antibodies against SARS-CoV-2, SARS-CoV-2 infection, COVID-19-related hospitalization, and COVID-19-related mortality. A meta-analysis will be conducted when more than one study per outcome is identified. The Higgins statistic (I^2^) was used to assess the heterogeneity of data from different studies. The random-effects model will be used when I^2^ ≥ 50%, otherwise, the fixed effect model will be adopted. For dichotomous data (e.g., seropositivity rates), the levels were presented as rates (%) with 95% confidential interval (CI). Comparisons between rates were presented as risk ratio (RR) with 95% CI. All outcomes will be presented as forest plots, if appropriate. Subgroup analyses and meta-regression were not carried out due to the low number of studies. The funnel plots and Harbord’s test were used to evaluate the potential publication bias. A two-sided *p*-value less than 0.05 was considered significant. Review Manager 5.4.1 and Stata 12.0 were used for statistical analysis.

## Results

### Study Inclusion

**FIGURE 1:**
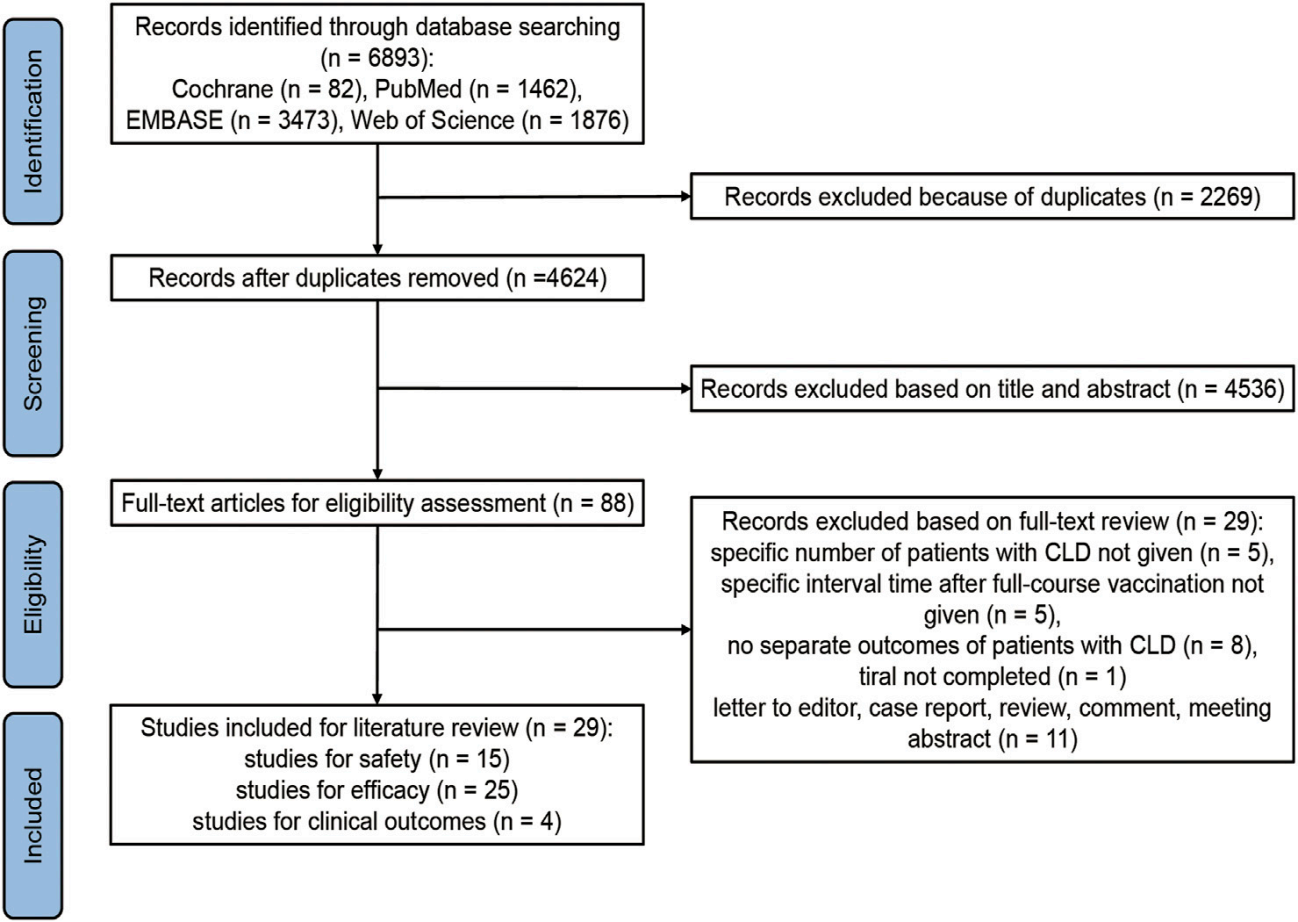
Flowchart summarizing the process for including the eligible studies. CLD, chronic liver disease (Global, 2022–2024).

6,893 records were identified through initial database searching, between which 2,269 records were removed records because of duplicates. Based on our inclusion and exclusion criteria, 4,536 records were excluded after title and abstract review, and further 29 records were excluded after full-text review. Ultimately, 29 studies were considered eligible and included in this literature review (Figure 1).

Of the 29 included studies [6, 18–45] (Table 1–3), 19 were prospective cohort studies, 8 was retrospective study, and 2 was cross-sectional study. In the 29 included studies, all patients were older than 18 years, and 17 studies included CLD patients with cirrhosis. 22 studies had a control group (18 studies used healthy people as controls and 4 study used unvaccinated CLD patients as controls). 11 studies included inactivated vaccines, 3 inactivated and viral vector vaccines, 2 viral vector vaccines, 4 mRNA and viral vector vaccines, 8 mRNA SARS-CoV-2 vaccines, and 1 mRNA, inactivated and viral vector vaccines. The follow-up time after full-course vaccination of the most included studies were more than 7 days. Overall, 25 studies evaluated the safety and/or antibody response of SARS-CoV-2 vaccines [18–21, 24–38, 40–45] (Tables 1, 2), 4 study evaluated the clinical outcome (SARS-CoV-2 infection, hospitalization and death) after full-course SARS-CoV-2 vaccination [6, 22, 23, 39] (Table 3). Besides, the risk of publication bias of all included studies was low or moderate (Supplementary Tables S2–S4).

**TABLE 1:** Characteristics of included studies on safety of SARS-CoV-2 vaccines (Global, 2022–2024).

Study	Country	Study design	Patients with liver disease (% with cirrhosis)	Age (years)	Controls	Vaccine (number of vaccinated patients, %), dose	Follow-up time (days) after full-course vaccination	Incidence of mild adverse events	Incidence of severe adverse events
Wang et al. [19]	China	Prospective cohort, multi-center study	381 with NAFLD (0% with cirrhosis)	Median 39.0 (IQR 33.0–48.0)	—	Inactivated (BBIBP-CorV, 381, 100%), 2 doses	At least 14, median 39.0 (IQR, 35.0–50.0)	29.4% within 28 days	0%
Thuluvath, et al. [20]	United States	Prospective cohort study	171 with CLD (46.2% with cirrhosis, 10 decompensated)	Mean 62.0	—	mRNA (Moderna, 77, 45.0%; Pfizer, 80, 46.8%), 2 doses; Viral vector (Johnson & Johnson, 14, 8.2%), 1 dose	At least 28, mean 40.8	Not available	0%
Ruether et al. [21]	Germany	Prospective cohort study	48 with CLD (100% with cirrhosis, 33.3% Child-Pugh class A, 37.5% Child-Pugh class B; 29.2% Child-Pugh class C)	Mean 53.8 (SD 9.5)	52 healthy adults matched by age and vaccination regimen	mRNA (Pfizer, 38, 79.2%; Moderna, 6, 12.4%), 2 doses; Viral vector (AZD1222, 1, 2.1%), 2 doses; AZD1222+mRNA (3, 6.3%), 2 doses	At least 10, median 28 (IQR, 21–41)	Dose 1 39.6% in patients and 30.8% in controls; Dose 2 37.5% in patients and 30.8% in controls	Dose 1 2.1% in patients and 7.7% in controls; Dose 2 6.3% in patients and 5.8% in controls*
Xiang et al. [18]	China	Cross-sectional study	149 with CHB (6.7% with compensated cirrhosis)	Median 41.0 (IQR 33.0–49.0)	—	Inactivated (BBIBP-CorV, CoronaVac, or WIBP-CorV, 149, 100%), 2 doses	At least 14, median 33 (IQR 24–48)	30.2% within 7 days	0%
Ai et al. [27]	China	Prospective cohort, multi-center study	437 with CLD (35.0% with cirrhosis, 123 compensated cirrhosis, 30 decompensated)	Median 47.0 (IQR 38.0–56.0)	144 healthy controls, age median 35.0 (IQR 28.5–41.5)	Inactivated (CoronaVac, BBIBP-CorV or WIBP-CorV, 437, 100%), 2 doses	At least 14	16% within 14 days of either dose	0.20%
He et al. [24]	China	Cross-sectional study	362 with CHB (13.3% with cirrhosis)	Median 45.0 (Range 19.0–78.0)	87 healthy adults matched by age, gender and BMI	Inactivated (BBIBP-CorV/CoronaVac, 362, 100%), 2 doses	At least 21 (Range 21–105)	14.1% in patients and 11.5% in controls within 30 days	0% in patients, 0% in controls within 30 days
Calleri et al. [25]	Italy	Prospective cohort study	89 with CLD (83.1% with cirrhosis)	Median 56.0 (IQR 50.0–62.0)	30 healthy controls, median age 55.0 (IQR 46.0–59.0)	mRNA (Pfizer, 83, 93.3%; Moderna 6, 6.7%), 88.8% completed 2 doses	Median 23 (IQR 14–42)	Not available	0% in patients, 0% in controls within 1 month
Bakasis et al. [26]	Greece	Prospective cohort study	87 with CLD [43.7% with cirrhosis, MELD: median 9 (IQR 6–25)]	Median 67.0 (Range 27.0–86.0)	40 healthy controls matched by age and gender	mRNA (Pfizer, 81, 93.1%; Moderna, 6, 6.9%), 2 doses	1 month	Not available	0% in patients, 0% in controls within 1 month
Biliotti et al. [44]	Italy	Prospective, single-center, observational study	149 cirrhotic patients (100% with cirrhosis, 133 Child-Pugh A, 16 Child-Pugh B/C)	Median 60 (IQR 55–64)	149 age and sex-matched HCWs	All cirrhotic patients: mRNA-1273 vaccine (Moderna); HCWs received the COVID-19 BNT162b2 vaccine (Pfizer-BioNTech) in 147 cases (98.7%) and the mRNA-1273 vaccine (Moderna) in 2 cases (1.3%)	1 month	101 (67.79) among patients with cirrhosis	0%
Chen et al. [43]	China	Prospective observational study	84 AILD (22.6% with cirrhosis)	Median 54.9 (IQR 49.3–60.8)	68 healthcare workers	Inactivated (BBIBP-CorV or Corona-Vac, 84, 100%), 2 doses	1 month (T1), 3 months (T2) and 6 months (T3)	26.2% in patients with AILD within 7 days	0%
Chen et al. [42]	China	Prospective observational study	192 severe liver disease (66% with cirrhosis)	Median 53 (47–59)	142 healthy controls and age median 48 (33–60)	Inactivated (BBIBP-CorV, 55, 29%; CoronaVac, 127, 66%; BBIBP-CorV + CoronaVac, 10, 5%), 2 doses	At least 21 days	33.3% in patients and 12.0% in controls within 7 days	0%
Li H. et al. [36]	China	Prospective observational study	76 with autoimmune liver disease (26.3% with cirrhosis)	Median 54.0 (IQR 48.8–60.2)	136 healthy controls age median 52.0 (33.0–62.2)	Inactivated (BBIBP-CorV: 21, 27.6%; CoronaVac: 49, 64.5%; BBIBP-CorV and CoronaVac: 6, 7.9%), 2, doses	At least 21 days	25.0%% in patients and 17.6%% in controls within 7 days	0%
Liu et al. [35]	China	Retrospective study	210 cirrhotic patients (100% with cirrhosis)	Mean 46.95 (5.45)	114 age‐matched vaccinated controls	Inactivated (CoronaVac, 210,100%), 2 doses	At least 14 days	26.2%% in patients and 20.2%% in controls within 7 days	0%
Ti et al. [32]	China	Retrospective and prospective epidemiological research	153 patients with CHB (0% with cirrhosis)	21∼68 (43.32 ± 12.65)	—	Inactivated vaccine, 153, 100%, 2 doses	At least 3 months	18.30% patients with CHB	0%
Wu et al. [29]	China	Prospective observational study	200 CHB (6% with cirrhosis)	Mean 47.39 ± 13.60	—	Inactivated (CoronaVac, 109, 2 doses); Viral vector (ZF2001, 91, 3 doses)	2 weeks	18.5% patients with CHB	0%

*Incidence of severe adverse events of dose 2 was used for meta-analysis. BMI, body mass index; CHB, chronic hepatitis B; CLD, chronic liver disease; IQR, interquartile range; NAFLD, non-alcoholic fatty liver disease; SD, standard deviation.

**TABLE 2:** Characteristics of included studies on antibody response of SARS-CoV-2 vaccines (Global, 2022–2024).

Study	Country	Study design	Patients with liver disease (% with cirrhosis)	Age (years)	Controls	Vaccine (number of vaccinated patients, %), dose	Follow-up time (days) after full-course vaccination	Seropositivity rates
Wang, J. et al. 2021 [19]	China	Prospective cohort, multi-center study	381 with NAFLD (0% with cirrhosis)	Median 39.0 (IQR 33.0–48.0)	—	Inactivated (BBIBP-CorV, 381, 100%), 2 doses	At least 14, median 39.0 (IQR, 35.0–50.0)	Neutralizing antibody, 95.5%
Thuluvath et al. [20]	United States	Prospective cohort study	171 with CLD (46.2% with cirrhosis, 10 decompensated)	Mean 62.0	—	mRNA (Moderna, 77, 45.0%; Pfizer, 80, 46.8%), 2 doses; Viral vector (Johnson & Johnson, 14, 8.2%), 1 dose	At least 28, mean 40.8	Anti-SARS-CoV-2 spike antibody, 95.9%
Ruether et al. [21]	Germany	Prospective cohort study	48 with CLD (100% with cirrhosis, 33.3% Child-Pugh class A, 37.5% Child-Pugh class B; 29.2% Child-Pugh class C)	Mean 53.8 (SD 9.5)	52 healthy adults matched by age and vaccination regimen	mRNA (Pfizer, 38, 79.2%; Moderna, 6, 12.4%), 2 doses; Viral vector (AZD1222, 1, 2.1%), 2 doses; AZD1222+mRNA (3, 6.3%), 2 doses	At least 10, median 28 (IQR, 21–41)	Anti-spike antibody 98% in patients and 100% in healthy controls; Anti-S RBD antibody 94% in patients and 100% in healthy controls
Xiang et al. [18]	China	Cross-sectional study	149 with CHB (6.7% with compensated cirrhosis)	Median 41.0 (IQR 33.0–49.0)	—	Inactivated (BBIBP-CorV, CoronaVac, or WIBP-CorV, 149, 100%), 2 doses	At least 14, median 33 (IQR 24–48)	Anti-S-RBD IgG, 87.25%; neutralizing antibody 74.5%
Ai et al. [27]	China	Prospective cohort, multi-center study	437 with CLD (35.0% with cirrhosis, 123 compensated cirrhosis, 30 decompensated)	Median 47.0 (IQR 38.0–56.0)	144 healthy controls, age median 35.0 (IQR 28.5–41.5)	Inactivated (CoronaVac, BBIBP-CorV or WIBP-CorV, 437, 100%), 2 doses	At least 14	Neutralizing antibody 77.3% in patients and 90.3% in healthy controls
He et al. [24]	China	Cross-sectional study	362 with CHB (13.3% with cirrhosis)	Median 45.0 (Range 19.0–78.0)	87 healthy adults matched by age, gender and BMI.	Inactivated (BBIBP-CorV/CoronaVac, 362, 100%), 2 doses	At least 21 (Range 21–105)	Anti-spike IgG 97.8% in patients and 100.0% in controls; Anti-RBD IgG 98.3% in patients and 100% in controls; Neutralizing antibody 72.6% in patients and 77.4% in controls
Calleri et al. [25]	Italy	Prospective cohort study	89 with CLD (83.1% with cirrhosis)	Median 56.0 (IQR 50.0–62.0)	30 healthy controls, median age 55.0 (IQR 46.0–59.0)	mRNA (Pfizer, 83, 93.3%; Moderna 6, 6.7%), 88.8% completed 2 doses	Median 23 (IQR 14–42)	Anti-spike IgG 94.9% in patients and 100% in controls
Bakasis et al. 2022 [26]	Greece	Prospective cohort study	87 with CLD [43.7% with cirrhosis, MELD: median 9 (IQR 6–25)]	Median 67.0 (Range 27.0–86.0)	40 healthy controls matched by age and gender	mRNA (Pfizer, 81, 93.1%; Moderna, 6, 6.9%), 2 doses	1 month	Anti-spike IgG 92.0% in patients and 100% in controls; Neutralizing antibody 89.7% in patients and 100% in controls
Al-Dury et al. [45]	Sweden	Prospective cohort study	48 with cirrhosis (100% with cirrhosis, 31 Child-Pugh A; 15 Child-Pugh B; 2 Child-Pugh C)	Median 63.5 (26–76)	39 healthy controls 60 (25–86)	mRNA (Moderna, 4, 8%; Pfizer-BioNTech, 44, 92%), 2 dose	6 months	Anti-RBD IgG 98% in patients and 100% in controls
Biliotti et al. [44]	Italy	Prospective, single-center, observational study	149 cirrhotic patients (100% with cirrhosis, 133 Child-Pugh A, 16 Child-Pugh B/C)	Median 60 (55–64)	149 age and sex-matched healthcare workers	All cirrhotic patients: mRNA-1273 vaccine (Moderna); HCWs received the COVID-19 BNT162b2 vaccine (Pfizer-BioNTech) in 147 cases (98.7%) and the mRNA-1273 vaccine (Moderna) in 2 cases (1.3%)	1 month	anti-S antibodies 100% in cirrhotic patients and HCWs
Chen et al. [43]	China	Prospective observational study	84 AILD (22.6% with cirrhosis)	Median 54.9 (49.3–60.8)	68 healthcare workers	Inactivated (BBIBP-CorV or Corona-Vac, 84, 100%), 2 doses	1 month (T1), 3 months (T2) and 6 months (T3)	Anti-RBD IgG 90% in patients and 100% in controls; Neutralizing antibody 90% in patients and 100% in controls
Chen et al. [42]	China	Prospective observational study	192 severe liver disease (66% with cirrhosis)	Median 53 (IQR 47–59)	142 healthy controls and age median 48 (IQR 33–60)	Inactivated (BBIBP-CorV, 55, 29%; CoronaVac, 127, 66%; BBIBP-CorV + CoronaVac, 10, 5%),2 doses	At least 21 days	Anti-RBD IgG 98.4% in patients and 100% in controls; Neutralizing antibody 57.8% in patients and 76.1% in controls
Duengelhoef et al. [41]	Germany	Prospective observational cohort study	112 consecutive patients with AIH (35% with cirrhosis) and 144 consecutive patients with cholestatic liver disease (17% with cirrhosis)	AIH 53 (17); PBC/PSC 52 (15)	95 healthy controls age 51 (8)	mRNA (BNT162b2; BioNTech SE/Pfizer or mRNA‐1273; Moderna Biotech); Viral vector vaccine (AZD1222; AstraZeneca). 2 doses	>2 weeks	Anti-spike antibody 98.2% in patients and 100% in healthy controls; Anti-RBD IgG 99.5% in patients and 100% in controls
Goel et al. [40]	India	Prospective observational cohort study	131 cirrhotic patients (61.1% with decompensated cirrhosis)	Median 50 (IQR 43–58)	—	Viral vector vaccine (AZD1222; AstraZeneca). 2 doses	4 weeks	Anti-spike antibody 99.2%% in patients; Neutralizing antibody 84% in patients
Kulkarni et al. [38]	India	Single-center prospective study	50 non-cirrhosis CLD and 113 Cirrhosis (69%)	NCCLD:49.34 ± 10.48; Cirrhosis: 52.42 ± 9.93	60 healthy controls age 51.2 ± 8.75	Viral vector vaccine (Covishield, 124, 76.07%); inactivated vaccines (Covaxin, 39, 23.93%), 2 dose	3 months	Anti-spike antibody 84.05% in patients and 91.7% in healthy controls
Li et al. [36]	China	Cross-sectional study with longitudinal follow-up	137 patients with liver dysfunction (47.5% with cirrhosis)	Mean 50.2	134 healthy controls and age mean 42.6	Inactivated:113, 82.5%, 2 doses; RBD-subunit recombinant: 24, 17.5%, 3 doses	At least 30 days	Neutralizing antibody 95.0%% in patients and 96.0% in healthy controls
Li et al. [36]	China	Prospective observational study	76 with autoimmune liver disease (26.3% with cirrhosis)	Median 54.0 (IQR 48.8–60.2)	136 healthy controls age median52.0 (IQR 33.0–62.2)	Inactivated (BBIBP-CorV: 21, 27.6%; CoronaVac: 49, 64.5%; BBIBP-CorV and CoronaVac: 6, 7.9%), 2, doses	At least 21 days	Anti-RBD IgG 97.4% in patients and 100% in controls; Neutralizing antibody 63.2% in patients and 84.6% in healthy controls
Liu et al. [34]	China	Prospective observational study	237 CLD (22.36% with cirrhosis)	Mean 47.01 (12.00)	170 healthy controls (HCs) of similar age and post-vaccination days	Inactivated (BBIBP-CorV; CoronaVac; WIBP-CorV)	At least 120 days	Anti-RBD IgG 87.34% in patients and 93.75% in controls; Neutralizing antibody 72.73%% in patients and 100%% in healthy controls
Singh et al. 2023 [33]	India	Retrospective study	88 Cirrhosis (15 CTP A, 71 CTP B and 2 CTP C)	Mean 53.3 ± 10.08	—	Viral vector (ChAdOx1-nCOV, 88, 100%), 2 doses	39 (23–76) days	Anti-spike antibody 92.05% in patients
Ti et al. [32]	China	Retrospective and prospective epidemiological research	153 patients with CHB (0% with cirrhosis)	21∼68 (43.32 ± 12.65)	—	Inactivated vaccine, 153, 100%, 2 doses	At least 3 months	Neutralizing antibody 45.50% in patients
Willauer et al. [31]	United States	Retrospective study	24 CLD (29% with cirrhosis)	Mean 61.0 ± 9.0	9 healthy controls and age 51.0 ± 14.5	mRNA (Pfizer/BioNTech (BNT162b2), 13 54%; Moderna (mRNA-1273) 11 46%), 2 doses	31 days (23–103)	Anti-spike antibody 95% in patients and 95.6% in healthy controls; Neutralizing antibody 95% in patients and 100% in healthy controls
Willuweit et al. [30]	Germany	Prospective observational study	110 Cirrhosis (69% Child A, 28% Child B and 3% Child C)	Median 55(IQR 45–66)	80 HCWs and age median 54 (IQR 45–59)	mRNA (BNT162b2 (Pfizer-BioNTech) 100%), 2 doses	69 days (43–106)	Anti-spike antibody 96% in patients and 99% in healthy controls
Wu et al. [29]	China	Prospective observational study	200 CHB (6% with cirrhosis)	Mean 47.39 ± 13.60	—	Inactivated (CoronaVac,109,2 doses); Viral vector (ZF2001, 91, 3 doses)	2 weeks	Neutralizing antibody 86.1%% in patients
Yang et al. 2023 [28]	China	Prospective multicenter study	261 chronic liver disease (79 compensated advanced CLD and 33 decompensated advanced CLD)	Non-ACLD: 38.0 (34.0, 47.0); CACLD: 55.0 (48.0, 59.0); DACLD: 54.0 (48.0, 59.0)	106 healthy controls and age median 46.0 (IQR 36.0, 54.8)	Inactivated (CoronaVac or BBIBP-CorV, 100%), 3, doses	6 months	Neutralizing antibody 73.18% in patients and 79.2% in healthy controls; Anti-spike antibody 77.39% in patients and 82.1% in healthy controls

**TABLE 3:** Characteristics of included studies on clinical outcomes of SARS-CoV-2 vaccines (Global, 2022–2024).

Study	Country	Study design	Patients with liver disease (% with cirrhosis)	Age (years)	Controls	Vaccine, dose (number of vaccinated patients, %)	Follow-up time (days) after full-course vaccination	SARS-CoV2 infection	Hospitalization for COVID-19	COVID-19 related death
John et al. [6]	United States	Retrospective cohort study	20,037 patients with cirrhosis (84.3% CTP A, 15.1% CTP B, 0.6% CTP C)	Median 69.1 (IQR 64.9–73.3)	20037 matched unvaccinated cirrhotic patients	mRNA vaccines (Pfizer/Moderna, 100%), 62.7% completed 2 doses	At least 7 days	0.03% in vaccinated patients and 0.14% in unvaccinated patients	0% in vaccinated patients and 0.02% in unvaccinated patients	0% in vaccinated patients and 0.01% in unvaccinated patients
John et al. [23]	United States	Retrospective cohort study	254 COVID-19 patients with cirrhosis (76.8% CTP A, 21.7% CTP B, 1.6% CTP C)	Median 63.8 (IQR 58.6–69.0)	508 matched unvaccinated COVID-19 patients with cirrhosis	mRNA vaccines (Pfizer, 126, 49.6%; Moderna, 121, 47.6%), 2 doses; Viral vector (Johnson & Johnson, 7, 2.8%), 1 dose. 32.3% completed full-course vaccination	At least 14 days	Not available	Not available	3.7% in vaccinated patients and 14.6% in unvaccinated patients
Moon et al. [22]	United States	Retrospective cohort study	21 COVID-19 patients with CLD (51% CTP A, 29% CTP B, 5% CTP C)	Median 59.0 (Range 28.0–72.0)	225 unvaccinated COVID-19 patients with CLD, median age 59.0	mRNA (Pfizer, 4, 19.0%; Moderna, 1, 4.8%), 2 doses; Inactivated (Bharat Biotech, 2, 9.5%; Sinovac, 1, 4.8%), 2 doses; Viral vector (Oxford-AZ, 12, 57.1%), 2 doses; Viral vector (Cansino, 1, 4.8%), 1 dose. 42.9% completed full-course vaccination	At least 14 days, median 21 days	Not available	33.3% in vaccinated patients and 72.0% in unvaccinated patients	0% in vaccinated patients and 8.0% in unvaccinated patients
Ivashkin et al. [39]	Russia	Retrospective cohort study	89 patients with cirrhosis (58.4% CTP A and 41.6% CTP B/C)	Median 59 (IQR 48–68)	148 matched unvaccinated cirrhotic patients	Viral vector: Gam-COVID-Vac (Sputnik V), 2 doses	At least 17 days	4.49% in vaccinated patients and 16.21% in unvaccinated patients	0% in vaccinated patients and 8.10% in unvaccinated patients	0% in vaccinated patients and 6.76% in unvaccinated patients

### Safety of SARS-CoV-2 Vaccination

Among the 15 studies reporting the safety of the SARS-CoV-2 vaccines, 12 had available results of MAEs, 15 had available results of SAEs, 5 had available results of MAEs of healthy controls, and 7 had available results of SAEs of healthy controls [18–21, 24–27, 29, 32, 35, 36, 42–44] (Table 1). In all 15 studies (2788 CLD patients), most adverse events were mild, and only six patients had SAEs (including local pain/swelling, fever, fatigue, headache, muscle pain, joint pain, diarrhea, and grade 3 ALT elevation) after SARS-CoV-2 vaccination. The results of meta-analysis showed that incidence of MAEs was 28.0% (95% CI 21.0%–36.0%) in CLD patients (Supplementary Table S5; Supplementary Figure S1A), and incidence of SAEs was 1.0% (95% CI 0%–27.0%) in CLD patients (Supplementary Table S5; Supplementary Figure S1B). Compared to healthy controls, CLD patients had higher incidence of MAEs (RR 1.60, 95% CI 1.27–2.02, *p* < 0.001) (Supplementary Figure S2A), while had similar incidence of SAEs (RR 1.08, 95% CI 0.23–5.11, *p* = 0.92) (Supplementary Figure S2B).

### Antibody Response of SARS-CoV-2 Vaccination

**FIGURE 2:**
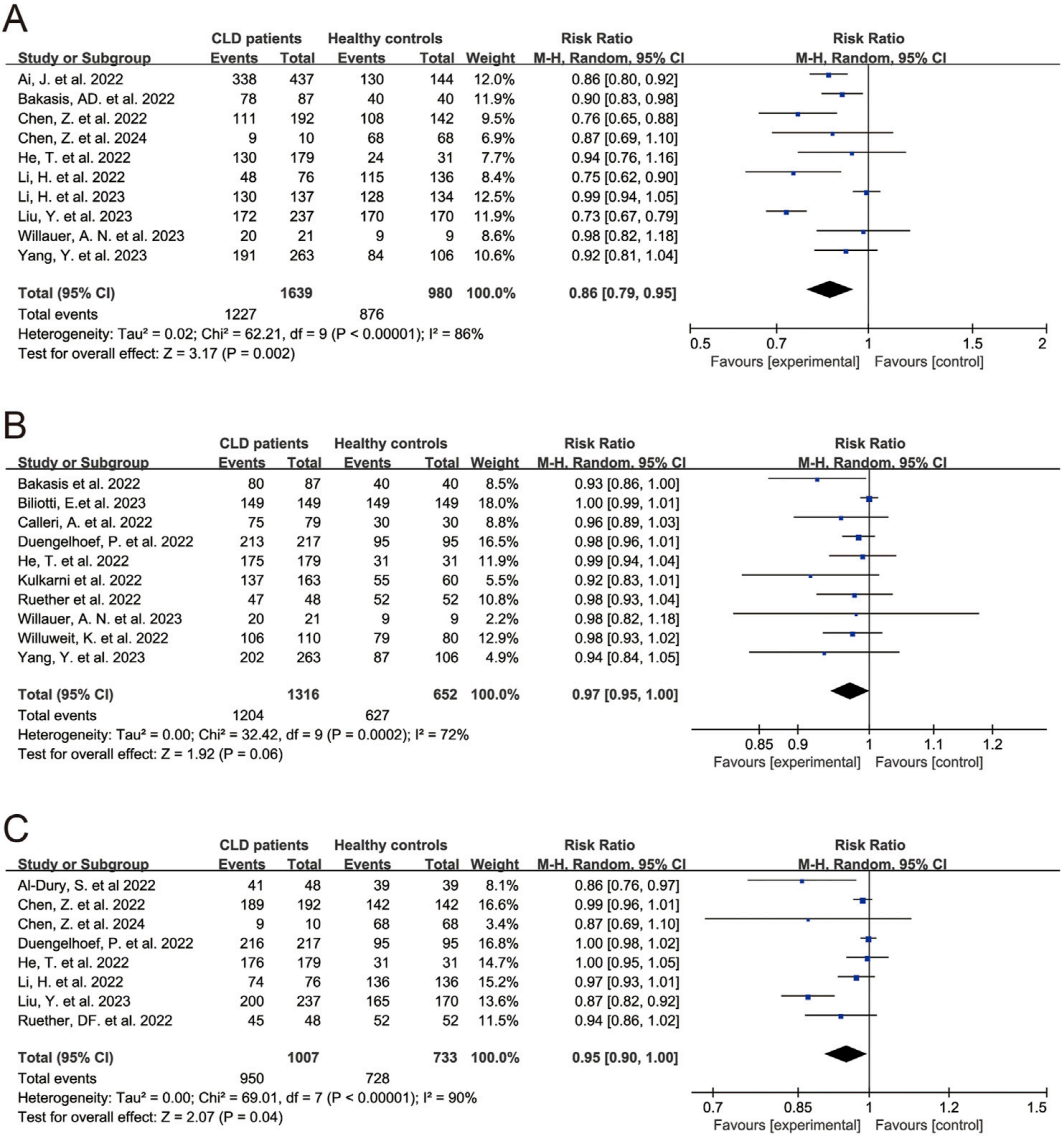
Forest plots of the comparison of the seropositivity rates of SARS-CoV-2 antibody between chronic liver disease patients and healthy controls. **(A)** Neutralizing antibody. **(B)** Anti-spike antibody. **(C)** Anti-receptor binding domain antibody. *p* < 0.05 was considered significant. CI, confidential interval; CLD, chronic liver disease; RBD, receptor binding domain (Global, 2022–2024).

In the 25 studies on the antibody response to SARS-CoV-2 vaccines, 5 determined the neutralizing antibody, 4 determined anti-spike antibody and neutralizing antibody, 2 determined anti-spike antibody and anti-receptor binding domain (RBD) antibody, 1 determined anti-RBD IgG, 7 determined anti-spike antibody, 5 determined neutralizing antibody and anti-RBD IgG, and 1 determined neutralizing antibody, anti-spike antibody and anti-RBD antibody [18–21, 24–38, 40–44] (Table 2). 18 studies had healthy controls. The results of meta-analysis showed seropositivity rates of neutralizing antibody, anti-spike antibody and anti-RBD antibody were 79.0% (95% CI 72.0%–87.0%), 94.0% (95% CI 91.0%–97.0%) and 96.0% (95% CI 93.0%–98.0%) in CLD patients, respectively (Supplementary Table S5). Compared to healthy controls, CLD patients had lower seropositivity rates of neutralizing antibody (RR 0.86, 95% CI 0.79–0.95, *p* = 0.002) (Figure 2A), anti-spike antibody (RR 0.97, 95% CI 0.95–1.00, *p* = 0.06) (Figure 2B) and anti-RBD antibody (RR 0.95, 95% CI 0.90–1.00, *p* = 0.04) (Figure 2C). Due to the fact that in evaluating the response of anti-spike antibody and anti-RBD antibody in patients with chronic liver disease after vaccination, some of the subjects in the literature were all patients with cirrhosis, we further conducted subgroup analysis, and the results remained unchanged (Supplementary Figures S3, S4).

### Clinical Outcome After SARS-CoV-2 Vaccination

**FIGURE 3:**
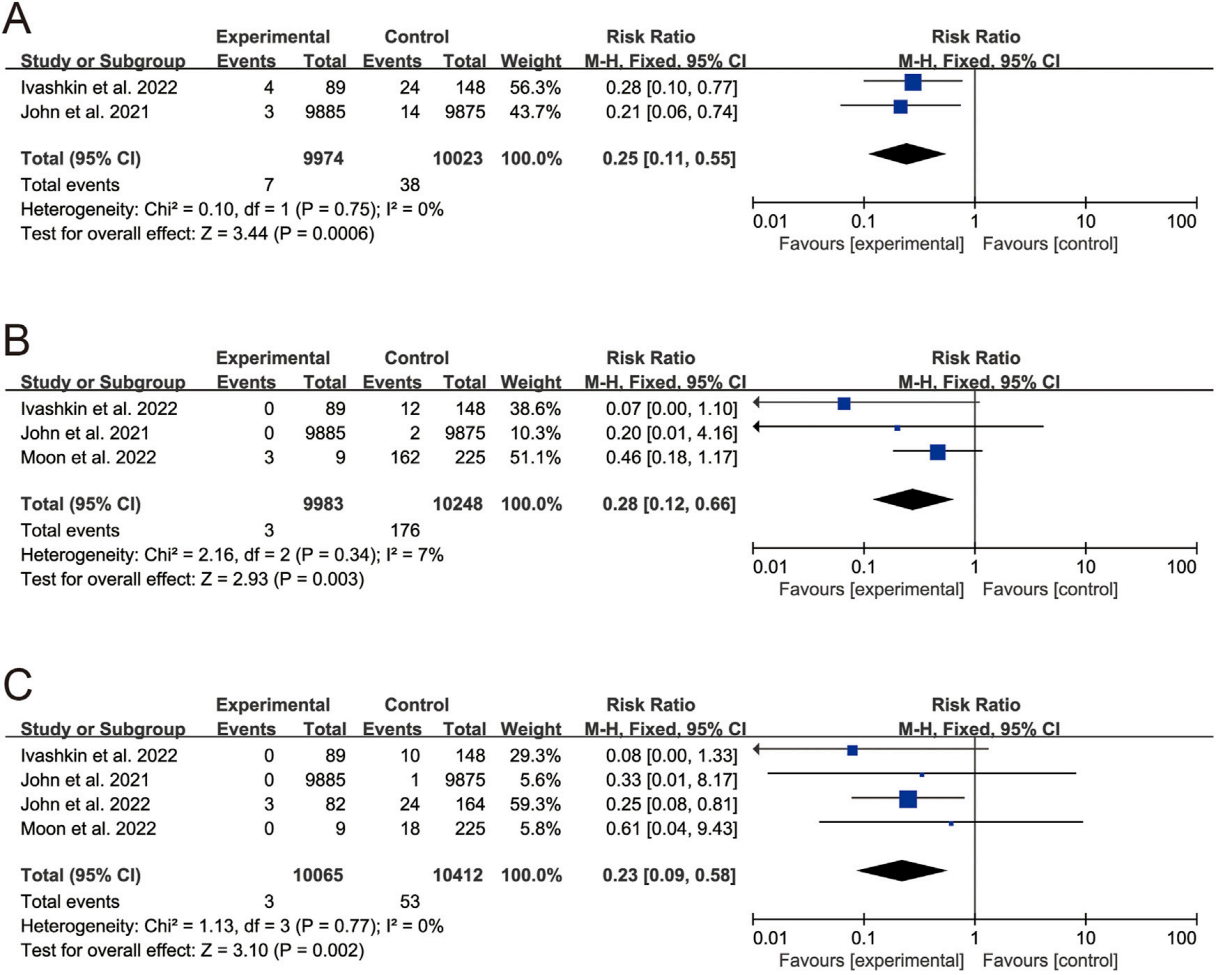
Forest plots of the comparison of the clinical outcome between vaccinated patients and unvaccinated patients. **(A)** SARS-CoV-2 infection. **(B)** COVID-19-related hospitalization. **(C)** COVID-19-related death. *p* < 0.05 was considered significant. CI, confidential interval; CLD, chronic liver disease (Global, 2022–2024).

Four study assessed the association between SARS-CoV-2 vaccination and clinical outcome [6, 22, 23, 39] (Table 3). The results indicated that, compared to unvaccinated CLD patients, CLD patients after full-course vaccination of SARS-CoV-2 vaccines had lower rates of SARS-CoV-2 infection (RR 0.25, 95% CI 0.11–0.55, *p* < 0.001) (Figure 3A), COVID-19-related hospitalization (RR 0.28, 95% CI 0.12–0.66, *p* = 0.003) (Figure 3B) and death (RR 0.23, 95% CI 0.09–0.58, *p* = 0.002) (Figure 3C).

### Publication Bias

The funnel plots showed no obvious asymmetry (Supplementary Figure S5), which indicated there might be no publication bias. Due to small number of eligible studies, only three outcomes (seropositivity rates of anti-spike antibody, neutralizing antibody, and COVID-19-related death) could be used to perform the Harbord’s test, and the result also indicated no publication bias (all *p* > 0.05) (Supplementary Table S6).

## Discussion

This systematic review and meta-analysis focused on the safety and efficacy of SARS-CoV-2 vaccines in patients with CLD. By analyzing the 29 eligible studies, SARS-CoV-2 vaccines were revealed to be safe in CLD patients. Full-course vaccination of SARS-CoV-2 vaccines induced promising antibody response (seropositivity rates of three antibodies were all higher than 80%) in CLD patients, but the seropositivity rates were lower in CLD patients than in healthy controls, which might decrease the immune protection provided by vaccination. Furthermore, full-course vaccination of SARS-CoV-2 vaccines may reduce the SARS-CoV-2 infection, COVID-19-related hospitalization and death in CLD patients.

The safety of SARS-CoV-2 vaccines is a highly concerned issue, and some previous studies reported thrombosis [46] and myocarditis cases [47] after SARS-CoV-2 vaccination. In this review, most AEs of CLD patients were mild, and the SAEs of CLD patients were rare. And the incidences of AEs were similar between CLD patients and HCs. Moreover, no thrombosis or myocarditis was reported. So, the results indicated good safety of SARS-CoV-2 vaccines in CLD patients.

CLD patients have dysregulated innate and adaptive immunity, which might weaken the immune response to vaccine [9]. In this review, the results of meta-analysis revealed that the seropositivity rates of SARS-CoV-2 antibody tended to be lower in CLD patients than in healthy controls, which indicated CLD might also weaken patients’ immune response to COVID-19 vaccine. Whereas, full-course vaccination of SARS-CoV-2 vaccines could still induce considerable antibody response in CLD patients (seropositivity rates of three antibodies were all higher than 80%). Furthermore, SARS-CoV-2 vaccination brought significant clinical benefit to CLD patients (vaccinated patients had significant lower proportion of SARS-CoV-2 infection, COVID-19-related hospitalization and death than that in unvaccinated patients). Therefore, SARS-CoV-2 vaccines had good efficacy in CLD patients.

Strengths of this study are as follows: first, this study was conducted following a pre-established protocol and guidelines, and different databases were used for including eligible studies, which helped to improve the quality of this study; Second, so far, there is no random controlled trial with large samples on CLD patients. In this context, this study is the first systematic review and meta-analysis on the safety and efficacy of SARS-CoV-2 vaccines in CLD patients, so it may provide relatively high-quality evidence for clinical practice. This study still has several limitations. First, due to lack of related data, this study did not assess the long-term efficacy of SARS-CoV-2 vaccines in CLD patients. Second, the sample of included studies were relatively small, and there was no random controlled trial (RCT) with large samples on CLD patients. Third, in the literature included in the meta-analysis, the subjects mainly had mild chronic liver disease, and no subgroup analysis was conducted for liver diseases of different severity levels. Forth, the recent emergence and global spreading of omicron subvariants have shown striking antibody evasion [48] and posed a critical challenge to the efficacy of SARS-CoV-2 vaccines. But until now, no study explored the efficacy of SARS-CoV-2 vaccines in CLD patients against omicron subvariants. So, there is a need for the studies on the long-term efficacy of SARS-CoV-2 vaccines and the efficacy of SARS-CoV-2 vaccines against omicron subvariants in CLD patients, and large-sample RCT.

In conclusion, SARS-CoV-2 vaccines showed good safety and efficacy in CLD patients. However, antibody response appeared to be lower in CLD patients than in healthy controls. Therefore, SARS-CoV-2 vaccines and booster doses should be given priority in this vulnerable population.

## Abbreviations

AHRQ, Agency for Healthcare Research and Quality; ALT, alanine aminotransferase; BMI, body mass index; CHB, chronic hepatitis B; CI, confidential interval; CLD, chronic liver disease; COVID-19, coronavirus disease 2019; IQR, interquartile range; MAEs, mild adverse events; MeSH, Medical Subject Headings; NAFLD, non-alcoholic fatty liver disease; OR, odds ratio; RBD, receptor binding domain; RCT, random controlled trial; RR, risk ratio; SAEs, serious adverse events; SARS-CoV-2, severe acute respiratory syndrome coronavirus 2; SD, standard deviation; WHO, world health organization.
