# Supplementary material for: Safety and Efficacy of SARS-CoV-2 Vaccines in Patients With Chronic Liver Diseases: A Systematic Review and Meta-Analysis
Source: Int J Public Health. 2024 Nov 21;69:1605295. doi: 10.3389/ijph.2024.1605295 (PMC11617177; doi:10.3389/ijph.2024.1605295)
Supplement: Supplementary file 1 [file DataSheet1.docx]

**Safety and efficacy of SARS-CoV-2 vaccines in patients with chronic liver diseases: a systematic review and meta-analysis**

Guanglin Xiao^#^, Taiyu He^#^, Biqiong Zhang^#^, Ziqiao Yang, Ning Ling, Min Chen, Dazhi Zhang, Peng Hu, Gaoli Zhang, Mingli Peng, Dachuan Cai, Hong Ren^*^

Table of contents

Supplementary Table 1.....................................................................................2

Supplementary Table 2.....................................................................................3

Supplementary Table 3.....................................................................................4

Supplementary Table 4.....................................................................................4

Supplementary Table 5.....................................................................................5

Supplementary Table 6.....................................................................................5

Supplementary Figure 1....................................................................................6

Supplementary Figure 2....................................................................................7

Supplementary Figure 3....................................................................................8

Supplementary Figure 4....................................................................................8

Supplementary Figure 5....................................................................................9

| **Table S1.** Search strategy | | | |
| --- | --- | --- | --- |
| **PubMed** | 2020 to 2024 | #1 | (hepatic diseases) OR (chronic liver diseases) OR (cirrhosis) OR (hepatitis) OR (NAFLD) OR (alcoholic liver disease) |
|  |  | #2 | "Liver Diseases"[Mesh] |
|  |  | #3 | #1 OR #2 |
|  |  | #4 | ((COVID-19) OR (SARS-CoV-2)) AND ((vaccine) OR (vaccination) OR (immunization)) |
|  |  | #5 | "COVID-19 vaccines"[Mesh] |
|  |  | #6 | #4 OR #5 |
|  |  | #7 | #3 and #6  (("Liver Diseases"[Mesh]) OR ((hepatic diseases) OR (chronic liver diseases) OR (cirrhosis) OR (hepatitis) OR (NAFLD) OR (alcoholic liver disease))) AND ((((COVID-19[Title/Abstract]) OR (SARS-CoV-2[Title/Abstract])) AND ((vaccine[Title/Abstract]) OR (vaccination[Title/Abstract]) OR (immunization[Title/Abstract]))) OR ("COVID-19 vaccines"[Mesh])) |
| **EMBASE** | 2020 to 2024 | #1 | 'hepatic diseases' OR (hepatic AND ('diseases'/exp OR diseases)) OR 'chronic liver diseases' OR (chronic AND ('liver'/exp OR liver) AND ('diseases'/exp OR diseases)) OR 'cirrhosis'/exp OR cirrhosis OR 'hepatitis'/exp OR hepatitis OR nafld OR 'alcoholic liver disease'/exp OR 'alcoholic liver disease' OR (('alcoholic'/exp OR alcoholic) AND ('liver'/exp OR liver) AND ('disease'/exp OR disease)) |
|  |  | #2 | 'liver disease'/exp |
|  |  | #3 | #1 or #2 |
|  |  | #4 | ('covid 19':ti,ab,kw OR 'sars cov 2':ti,ab,kw) AND (vaccine:ti,ab,kw OR vaccination:ti,ab,kw OR immunization:ti,ab,kw) |
|  |  | #5 | 'sars-cov-2 vaccine'/exp |
|  |  | #6 | #4 or #5 |
|  |  | #7 | #3 and #6 AND [humans]/lim AND [2020-2022]/py |
| **Cochrane Central Register of Controlled Trials (CENTRAL) in the Cochrane Library** | 2020 to 2024 | #1 | ((hepatic diseases) OR (chronic liver diseases) OR (cirrhosis) OR (hepatitis) OR (NAFLD) OR (alcoholic liver disease)) |
|  |  | #2 | MeSH descriptor: [Liver Diseases] explode all trees |
|  |  | #3 | #1 or #2 |
|  |  | #4 | (((COVID-19) OR (SARS-CoV-2)) AND ((vaccine) OR (vaccination) OR (immunization))):ti,ab,kw |
|  |  | #5 | MeSH descriptor: [COVID-19 vaccines] explode all trees |
|  |  | #6 | #4 or #5 |
|  |  | #7 | #3 and #6 |
| **Web of Science** | 2020 to 2024 | #1 | (hepatic diseases) OR (chronic liver diseases) OR (cirrhosis) OR (hepatitis) OR (NAFLD) OR (alcoholic liver disease) (All Fields) or Liver Diseases (All Fields) |
|  |  | #2 | ((COVID-19) OR (SARS-CoV-2)) AND ((vaccine) OR (vaccination) OR (immunization)) (Topic) or COVID-19 vaccines (Topic) |
|  |  | #3 | #1 and #2 |

| **Table S2. Quality assessment of included studies based on the Newcastle-Ottawa scale** | | | | | | | | | |
| --- | --- | --- | --- | --- | --- | --- | --- | --- | --- |
|  | **Selection** | | | | **Comparability** | **Outcome** | | |  |
| **Study** | **Representativeness of the exposed cohort** | **Selection of the non-exposed cohort** | **Ascertainment of exposure** | **Demonstration that outcome of interest was not present at start of study** | **Comparability of cohorts on the basis of the design or analysis** | **Assessment of outcome** | **Was follow-up long enough for outcomes to occur** | **Adequacy of follow up of cohorts** | **Total** |
| Wang, J. et al. 2021 | * |  | * | * |  | * | * | * | 6 |
| Thuluvath, PJ. et al. 2021 | * |  | * | * | * | * | * | * | 7 |
| Ruether, DF. et al. 2022 | * |  | * | * | * | * | * | * | 7 |
| Ai, J. et al. 2022 | * | * | * | * |  | * | * | * | 7 |
| John, BV, et al.2021 | * | * | * | * | ** | * | * | * | 9 |
| Calleri, A. et al. 2022 | * |  | * | * | * | * | * | * | 7 |
| Bakasis, AD. et al. 2022 | * | * | * | * | ** | * | * | * | 9 |
| John, BV, et al.2022 | * | * | * | * | ** | * | * | * | 9 |
| Moon, AM, et al. 2021 | * | * | * | * | * | * | * | * | 8 |
| Biliotti, E.et al. 2023 | * | * | * | * | * | * | * | * | 8 |
| Chen, Z. et al. 2024 | * | * | * | * | * | * | * | * | 8 |
| Chen, Z. et al. 2022 | * | * | * | * | * | * | * | * | 8 |
| Al-Dury, S. et al 2022 | * | * | * | * | * | * | * | * | 8 |
| Duengelhoef, P. et al. 2022 | * | * | * | * | ** | * | * | * | 9 |
| Goel, A. et al. 2022 | * |  | * | * | * | * | * | * | 7 |
| Kulkarni, A. V. et al. 2022 | * | * | * | * | ** | * | * | * | 9 |
| Li, H. et al. 2023 | * | * | * | * |  | * | * | * | 7 |
| Li, H. et al. 2022 | * | * | * | * |  | * | * | * | 7 |
| Liu, F. et al. 2023 | * | * | * | * | * | * | * | * | 8 |
| Liu, Y. et al. 2023 | * | * | * | * | * | * | * | * | 8 |
| Singh, A. et al. 2023 | * |  | * | * |  | * | * | * | 6 |
| Ti, Y. N. et al. 2022 | * | * | * | * | * | * | * | * | 8 |
| Willuweit, K. et al. 2022 | * | * | * | * |  | * | * | * | 7 |
| Willauer, A. N. et al. 2023 | * | * | * | * | * | * | * | * | 8 |
| Wu, S. et al. 2023 | * |  | * | * |  | * | * | * | 6 |
| Yang, Y. et al. 2023 | * | * | * | * | * | * | * | * | 8 |
| Ivashkin, V. et al. 2022 | * | * | * | * | ** | * | * | * | 9 |

| **Table S3. Cross-Sectional Study Quality for study of Xiang et al.** | | | |
| --- | --- | --- | --- |
| **Item** | **Yes** | **No** | **Unclear** |
| 1) Define the source of information (survey, record review) | √ |  |  |
| 2) List inclusion and exclusion criteria for exposed and unexposed subjects (cases and controls) or refer to previous publications | √ |  |  |
| 3) Indicate time period used for identifying patients | √ |  |  |
| 4) Indicate whether or not subjects were consecutive if not population-based |  |  | √ |
| 5) Indicate if evaluators of subjective components of study were masked to other aspects of the status of the participants |  |  | √ |
| 6) Describe any assessments undertaken for quality assurance purposes (e.g., test/retest of primary outcome measurements) |  | √ |  |
| 7) Explain any patient exclusions from analysis |  | √ |  |
| 8) Describe how confounding was assessed and/or controlled. |  | √ |  |
| 9) If applicable, explain how missing data were handled in the analysis | √ |  |  |
| 10) Summarize patient response rates and completeness of data collection | √ |  |  |
| 11) Clarify what follow-up, if any, was expected and the percentage of patients for which incomplete data or follow-up was obtained | √ |  |  |
| Total Score | 6 | | |

| **Table S4. Cross-Sectional Study Quality for study of He et al.** | | | |
| --- | --- | --- | --- |
| **Item** | **Yes** | **No** | **Unclear** |
| 1) Define the source of information (survey, record review) | √ |  |  |
| 2) List inclusion and exclusion criteria for exposed and unexposed subjects (cases and controls) or refer to previous publications | √ |  |  |
| 3) Indicate time period used for identifying patients | √ |  |  |
| 4) Indicate whether or not subjects were consecutive if not population-based |  |  | √ |
| 5) Indicate if evaluators of subjective components of study were masked to other aspects of the status of the participants |  |  | √ |
| 6) Describe any assessments undertaken for quality assurance purposes (e.g., test/retest of primary outcome measurements) | √ |  |  |
| 7) Explain any patient exclusions from analysis |  |  | √ |
| 8) Describe how confounding was assessed and/or controlled. | √ |  |  |
| 9) If applicable, explain how missing data were handled in the analysis | √ |  |  |
| 10) Summarize patient response rates and completeness of data collection | √ |  |  |
| 11) Clarify what follow-up, if any, was expected and the percentage of patients for which incomplete data or follow-up was obtained | √ |  |  |
| Total Score | 8 | | |

| **Table S5. Summary of safety and seropositivity rates of eligible studies** | | | | | |
| --- | --- | --- | --- | --- | --- |
|  | **Mild adverse events** | **Severe adverse events** | **Neutralizing antibody** | **Anti-spike antibody** | **Anti-RBD antibody** |
| Number of eligible studies for analysis | 12 | 2 | 15 | 13 | 9 |
| Incidence/Seropositivity rates | 28.0%, 95% CI 21.0%-36.0% | 1.0%, 95% CI 0%-27.0% | 79.0%, 95% CI 72.0%-87.0% | 94.0%, 95% CI 91.0%-97.0% | 96.0%, 95% CI 93.0%-98.0% |
|  | | | | | |

| **Table S6. Publication bias of safety and seropositivity rates of eligible studies** | | | | | | | | |
| --- | --- | --- | --- | --- | --- | --- | --- | --- |
|  | **Mild adverse events** | **Severe adverse events** | **Anti-spike antibody** | **Anti-RBD antibody** | **Neutralizing antibody** | **SARS-CoV-2 infection** | **COVID-19-related hospitalization** | **COVID-19-related death** |
| Number of eligible studies for Harbord’s test | 5 | 1 | 10 | 8 | 10 | 2 | 3 | 4 |
| P values of Harbord’s test | 0.272 | No applicable | 0.488 | 0.892 | 0.455 | No applicable | 0.660 | 0.443 |
| *p* < 0.05 was considered statistically significant. RBD, receptor binding domain | | | | | | | | |


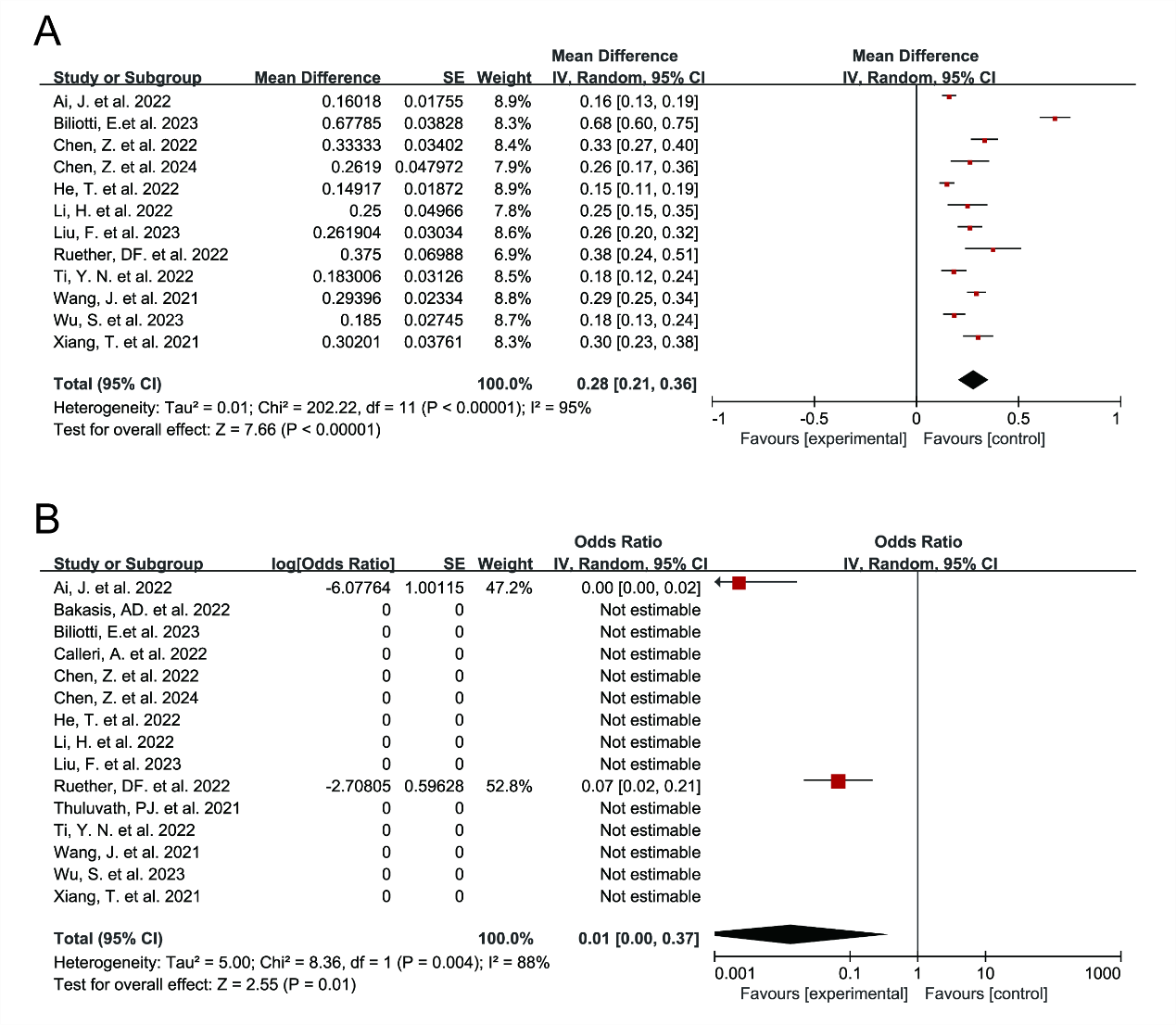


**Figure S1.** Forest plots of the incidence of adverse events in CLD patients. **(A)** Mild adverse events. **(B)** Severe adverse events. To obtain the incidence of severe adverse events, conversion calculation needed to be done as follows: P (incidence) = OR/(1+OR), 95%CI LL= LLOR/(1+LLOR), 95%CI UL=ULOR/(1+ULOR). CI, confidential interval; CLD, chronic liver disease; LL, lower limit; OR, odds ratio; SE, standard error; UL, upper limit.


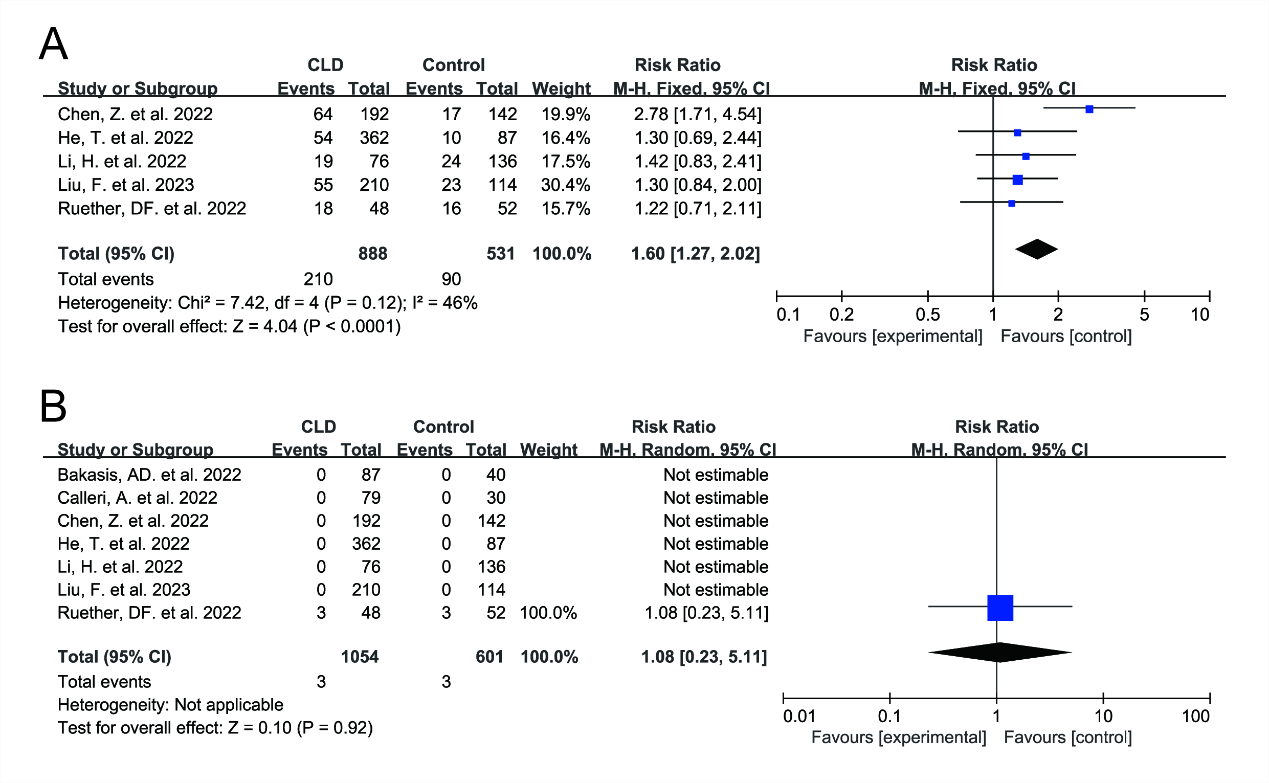


**Figure S2.** Forest plots of the comparison of the incidence of adverse events between CLD patients and healthy controls. **(A)** Mild adverse events. **(B)** Severe adverse events. *p* < 0.05 was considered significant. CI, confidential interval; CLD, chronic liver disease.


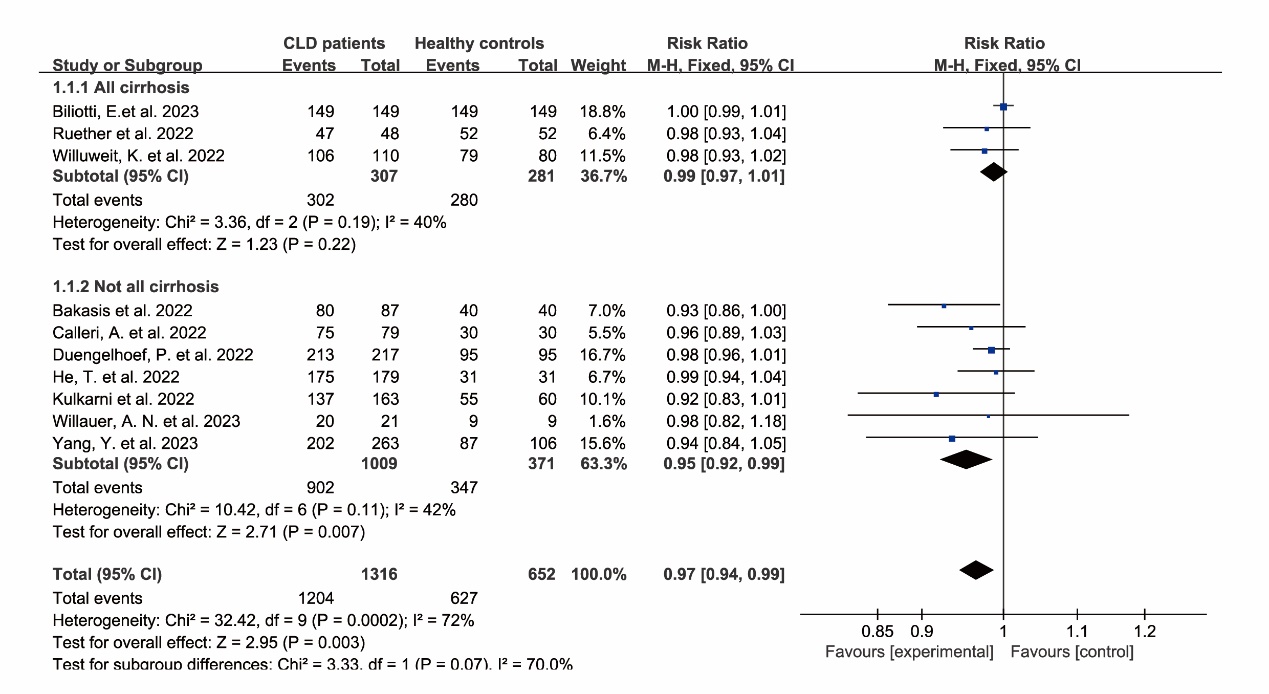


**Figure S3**. Comparison of anti-spike antibody seropositivity rates in CLD patients vs. healthy controls: subgroup analysis by cirrhosis. p < 0.05 was considered significant. CI, confidential interval; RBD, receptor binding domain.

**
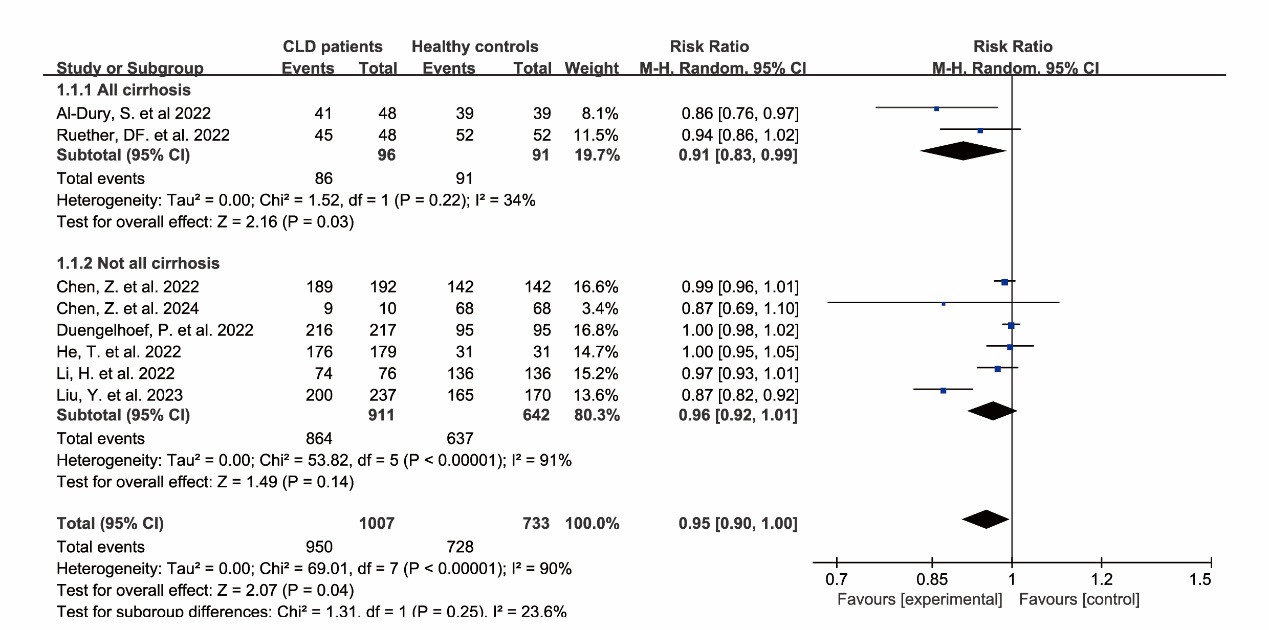
**

**Figure S4.** Comparison of anti-RBD antibody seropositivity rates in CLD patients vs. healthy controls: subgroup analysis by cirrhosis. p < 0.05 was considered significant. CI, confidential interval; RBD, receptor binding domain.


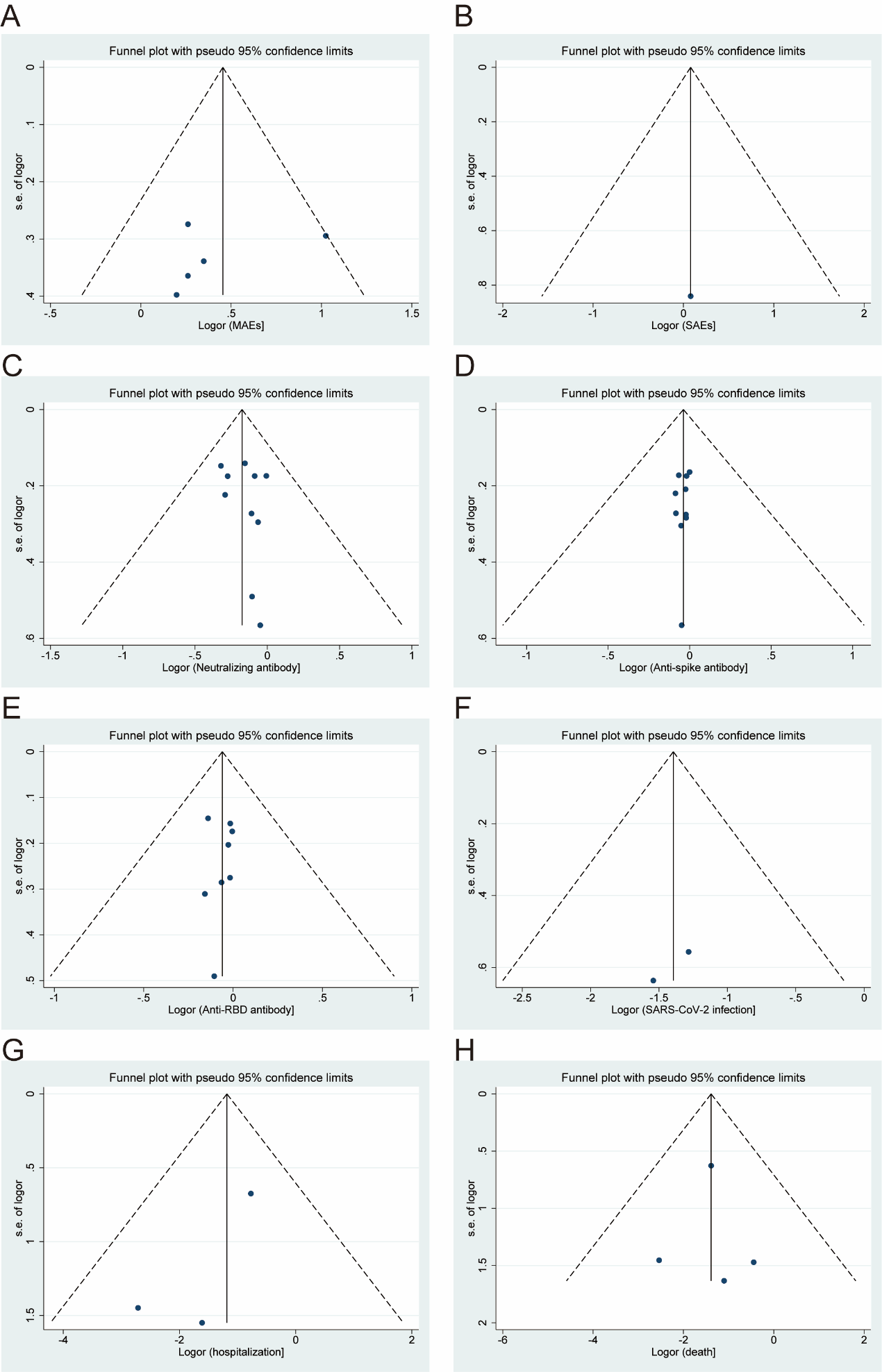


**Figure S5.** Funnel plots of outcomes of eligible studies. Funnel plots of **(A)** mild adverse events, **(B)** severe adverse events, **(C)** neutralizing antibody, **(D)** anti-spike antibody, **(E)** anti-RBD antibody, **(F)** SARS-CoV-2 infection, **(G)** COVID-19-related hospitalization, **(H)** COVID-19-related death of studies containing both CLD patients and healthy controls. CLD, chronic liver disease; MAEs, mild adverse events; OR, odds ratio; RBD, receptor binding domain; SAEs, severe adverse events.
